# Synovial Fluid Fatty Acid Profiles Are Differently Altered by Inflammatory Joint Pathologies in the Shoulder and Knee Joints

**DOI:** 10.3390/biology10050401

**Published:** 2021-05-04

**Authors:** Anne-Mari Mustonen, Reijo Käkelä, Antti Joukainen, Petri Lehenkari, Antti Jaroma, Tommi Kääriäinen, Heikki Kröger, Tommi Paakkonen, Sanna P. Sihvo, Petteri Nieminen

**Affiliations:** 1 Institute of Biomedicine, School of Medicine, Faculty of Health Sciences, University of Eastern Finland, P.O. Box 1627, FI-70211 Kuopio, Finland; tommi.paakkonen@uef.fi (T.P.); petteri.nieminen@uef.fi (P.N.); 2 Department of Environmental and Biological Sciences, Faculty of Science and Forestry, University of Eastern Finland, P.O. Box 111, FI-80101 Joensuu, Finland; 3 Molecular and Integrative Biosciences Research Programme, Faculty of Biological and Environmental Sciences, University of Helsinki, P.O. Box 65, FI-00014 Helsinki, Finland; reijo.kakela@helsinki.fi (R.K.); sanna.sihvo@helsinki.fi (S.P.S.); 4 Helsinki University Lipidomics Unit (HiLIPID), Helsinki Institute for Life Science (HiLIFE) and Biocenter Finland, University of Helsinki, P.O. Box 65, FI-00014 Helsinki, Finland; 5 Pohjola Hospital, Leväsentie 1, FI-70700 Kuopio, Finland; antti.joukainen@pohjolasairaala.fi (A.J.); tommi.kaariainen@pohjolasairaala.fi (T.K.); 6 Cancer and Translational Medicine Research Unit, Faculty of Medicine, University of Oulu, P.O. Box 5000, FI-90014 Oulu, Finland; petri.lehenkari@oulu.fi; 7 Department of Surgery and Medical Research Center, Oulu University Hospital, P.O. Box 21, FI-90029 OYS, Finland; 8 Department of Orthopaedics, Traumatology and Hand Surgery, Kuopio University Hospital, P.O. Box 100, FI-70290 Kuopio, Finland; Antti.Jaroma@kuh.fi (A.J.); Heikki.Kroger@kuh.fi (H.K.)

**Keywords:** fatty acid, knee, n-3 polyunsaturated fatty acids, n-6 polyunsaturated fatty acids, osteoarthritis, rheumatoid arthritis, shoulder, synovial fluid

## Abstract

**Simple Summary:**

Anomalies of fatty acid metabolism characterize osteoarthritis and rheumatoid arthritis in the knee joint. No previous study has investigated the synovial fluid fatty acid manifestations in these aging-related inflammatory diseases in the shoulder. The present experiment compared the fatty acid alterations between the shoulder and knee joints in trauma controls and in patients with end-stage osteoarthritis or end-stage rheumatoid arthritis. The fatty acid signatures in the synovial fluid of trauma controls were mostly uniform in both anatomical locations. Shoulders with rheumatoid arthritis were characterized by elevated percentages of arachidonic acid and docosahexaenoic acid and with reduced proportions of oleic acid. The fatty acid profiles of knees with osteoarthritis or rheumatoid arthritis were relatively uniform and displayed lower proportions of linoleic acid, docosahexaenoic acid and total n-6 polyunsaturated fatty acids. The results indicate location- and disease-dependent differences in the synovial fluid fatty acid composition. These alterations may affect joint lubrication, synovial inflammation and pannus formation as well as cartilage and bone degradation and contribute to the pathogeneses of inflammatory joint diseases.

**Abstract:**

Anomalies of fatty acid (FA) metabolism characterize osteoarthritis (OA) and rheumatoid arthritis (RA) in the knee joint. No previous study has investigated the synovial fluid (SF) FA manifestations in these aging-related inflammatory diseases in the shoulder. The present experiment compared the FA alterations between the shoulder and knee joints in patients with end-stage OA or end-stage RA. SF samples were collected during glenohumeral or knee joint surgery from trauma controls and from OA and RA patients (n = 42). The FA composition of SF total lipids was analyzed by gas chromatography with flame ionization and mass spectrometric detection and compared across cohorts. The FA signatures of trauma controls were mostly uniform in both anatomical locations. RA shoulders were characterized by elevated percentages of 20:4n-6 and 22:6n-3 and with reduced proportions of 18:1n-9. The FA profiles of OA and RA knees were relatively uniform and displayed lower proportions of 18:2n-6, 22:6n-3 and total n-6 polyunsaturated FAs (PUFAs). The results indicate location- and disease-dependent differences in the SF FA composition. These alterations in FA profiles and their potential implications for the production of PUFA-derived lipid mediators may affect joint lubrication, synovial inflammation and pannus formation as well as cartilage and bone degradation and contribute to the pathogeneses of inflammatory joint diseases.

## 1. Introduction

Free fatty acids (FAs) are potential mediators of inflammation and cartilage and bone destruction in aging-related joint diseases such as osteoarthritis (OA) and rheumatoid arthritis (RA) [1,2]; the latter is autoimmune-driven and characterized by a higher inflammatory load [3]. Obesity, a recognized risk factor for OA due to altered joint loading and chronic low-grade systemic inflammation [4], is associated with elevated levels of circulating free FAs [5] and ratios of n-6 to n-3 polyunsaturated FAs (PUFAs) [6]. FA profiles are also altered in circulation, synovial fluid (SF) and joint tissues in OA and RA patients [7,8,9,10,11] and the production of PUFA-derived lipid mediators (oxylipins) including specialized pro-resolving mediators (SPMs) may be higher in RA than in OA [3,12].

Individual FAs can induce diverse effects on chondrocytes, synoviocytes, osteoblasts and osteoclasts [13]. Saturated FAs (SFAs), palmitic and stearic acid (16:0, 18:0), have a mostly deleterious influence on joint tissues as they can induce endoplasmic reticulum stress, the production of cytokines and cartilage-degrading proteinases and chondrocyte apoptosis [14,15,16]. In contrast, long-chain n-3 PUFAs, especially eicosapentaenoic acid (20:5n-3, EPA), can have positive effects on synovium, cartilage and bone health [17,18,19]. The situation can be more complex for n-6 PUFAs such as linoleic acid (18:2n-6, LA), arachidonic acid (20:4n-6, ARA) and its derivative prostaglandin E_2_ (PGE_2_) displaying a combination of potentially adverse and beneficial effects on cytokine production, pannus formation, cartilage degradation and bone resorption [2,19,20,21,22,23]. Regarding dietary FAs, the consumption of SFAs and n-6 PUFAs has been associated with an increased risk of developing OA whereas n-3 PUFAs and monounsaturated FAs (MUFAs) may induce protective effects [24,25]. In addition, a high intake of long-chain n-3 PUFAs may protect from the development of RA and improve pain symptoms, tender joint count and the duration of morning stiffness as well as decrease the consumption of non-steroidal anti-inflammatory drugs [26]. However, the situation is not straightforward and inconsistent data also exist on the influence of individual FAs [17,23], emphasizing the complexity of the effects induced by FAs and their derivatives on the degradation and protection of synovial joints. Moreover, the biological relevance of in vitro studies using a single FA can be questioned as in vivo the joint tissue FA profiles are composed of a complex FA mixture.

The glenohumeral joint is a non-weight-bearing joint with a large range of motion and its stability mainly depends on active muscle control with only minor roles for the glenohumeral capsule, labrum and ligaments [27,28]. OA and RA in the shoulder have been studied less than these diseases in the knee even though they can cause significant pain and disability [29,30]. There are differences in the anatomy and histology of these two synovial joints. The humeral head has thinner articular cartilage compared with the femoral condyle [31]. A special feature of the shoulder joint is that early degenerative changes can be present in the extracellular matrix of the humeral head and glenoid cartilage at a normal state [32]. Biochemically, proteoglycan synthesis is similar in the humeral head, glenoid and femoral cartilages. When compared with the femoral condyle/tibial plateau/patella, the levels of chondroitin sulfate were lower and those of collagen higher in the normal articular cartilage of the humeral head [33]. Little is known about the potential differences in lipid metabolism between these joints in the healthy state let alone in joint pathologies. Due to their ubiquitous nature and relatively easy analytics, FAs could be provisionally good candidates for SF biomarkers of inflammatory joint diseases.

There is an urgent need for biomarkers to detect especially early OA before radiological imaging can discern the condition and irreversible joint damage has taken place. It is known, for instance, that the levels of catabolic biomarkers such as sulfated glycosaminoglycan can increase in shoulder SF during joint degradation [34]. In the present study, we aimed (i) to investigate the modifications in FA signatures in OA and RA SF and (ii) to compare the FA profiles between diseased shoulder and knee joints. It was hypothesized that (i) RA SF would be more inflammatory in its FA composition and that (ii) there would be differences in the inflammation-related FA alterations between the two investigated joints as the cartilage lining of the shoulder joint is not as susceptible to mechanical wear and tear as that of the knee joint.

## 2. Materials and Methods

### 2.1. Patients and Sampling

SF samples were collected during arthroscopic (e.g., debridement, ligament and tendon repair) or joint replacement surgery from patients with shoulder (n = 17) or knee joint disorders (n = 25) operated on at the Oulu or Kuopio University Hospital. The study was approved by the Ethical Committees of the hospitals (Oulu: decision #29/2011, amendment 2/24/2014; Kuopio: decision #79//2013) in compliance with the Helsinki Declaration and, prior to the surgery, all participants provided written informed consent to donate their SF samples. Only patients ≥ 18 years old were sampled. They were surveyed for general data (gender, age, body mass, height, body mass index (BMI), operation, operative diagnosis and medication) (Table 1). SF samples were collected with sterile needles and syringes and stored at −70 °C until analyzed. The joints were categorized as trauma controls without a history of arthritis (n = 16), end-stage OA (n = 17) and end-stage RA (n = 9).

### 2.2. Fatty Acid Analysis

Subsamples of SF (50–75 μL) were transmethylated in methanolic H_2_SO_4_ under a nitrogen atmosphere and the formed FA methyl esters (FAMEs) were extracted with hexane and analyzed by a Shimadzu GC-2010 Plus gas chromatograph (Shimadzu, Kyoto, Japan) as previously outlined [10]. The FAME structures were confirmed by using electron impact mass spectra recorded by a Shimadzu GCMS-QP2010 Ultra with a mass selective detector. The results represented the FA composition (mol-%) in SF total lipids. Parts of knee FA data were included in our previous paper [10]. The PUFA product/precursor ratios, double bond index (DBI) and total average chain length (TACL) were calculated as previously described [10].

### 2.3. Statistical Analyses

Statistical comparisons between the study groups were performed with the generalized linear model (IBM SPSS v25 software, IBM, Armonk, NY, USA). Sex ratios were tested with the Fisher’s exact test. Correlations were calculated with the Spearman correlation coefficient (r_s_). The *p* value < 0.05 was considered statistically significant. The results were presented as the mean ± SE. We also performed the discriminant analysis by classifying the FA data by discriminant functions to see how clearly the samples in the study groups differed from one another, which variables separated them most clearly and how well the analysis was able to classify the samples into their respective study groups. To validate the model, the knee OA patients were divided into two study populations. The Kuopio subsample was first analyzed separately and then together with the Oulu subsample to assess whether all OA knees would cluster together based on their FA profiles.

## 3. Results

**Figure 1:**
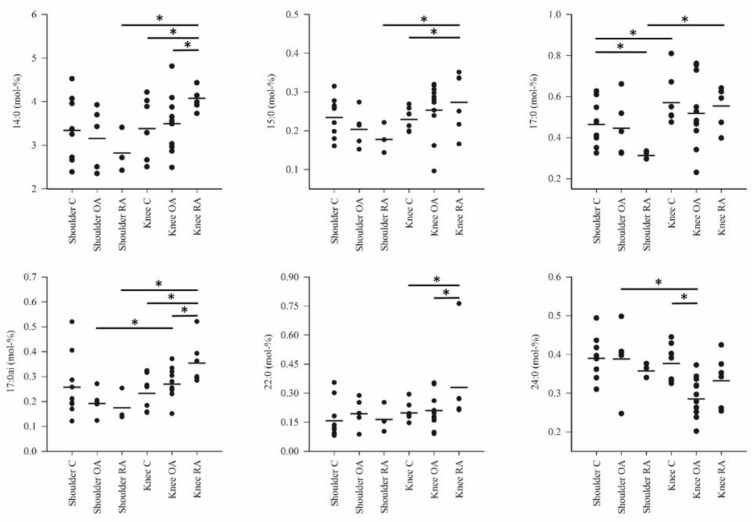
The percentages (mol-%; individual values and means) of selected saturated fatty acids in the synovial fluid samples. C = control, OA = osteoarthritis, RA = rheumatoid arthritis. * = significant difference between the two study groups at the ends of the connecting line (*p* < 0.05).

**Figure 2:**
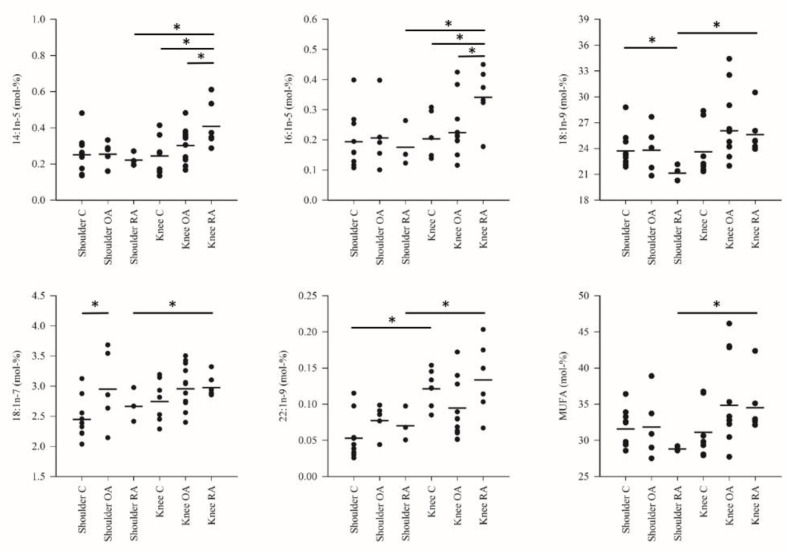
The percentages (mol-%; individual values and means) of selected monounsaturated fatty acids (MUFA) and their sum in the synovial fluid samples. C = control, OA = osteoarthritis, RA = rheumatoid arthritis. * = significant difference between the two study groups at the ends of the connecting line (*p* < 0.05).

**Figure 3:**
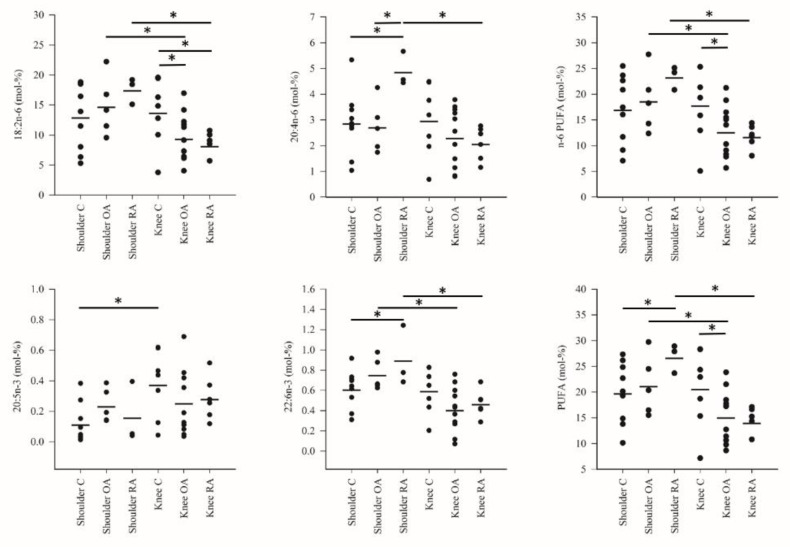
The percentages (mol-%; individual values and means) of selected polyunsaturated fatty acids (PUFA) and their sums in the synovial fluid samples. C = control, OA = osteoarthritis, RA = rheumatoid arthritis. * = significant difference between the two study groups at the ends of the connecting line (*p* < 0.05).

The differences in the FA profiles between the study groups are represented in Figure 1, Figure 2 and Figure 3 and Supplementary Table S1. The FA alterations that characterized the baseline differences between the control shoulders and the control knees were minor: higher percentages of 17:0, EPA and 22:1n-9 in knee SF were the only statistically significant differences. Regarding shoulder OA, increased 18:1n-7 proportions were documented in comparison with the shoulder controls. Shoulder RA differed from the corresponding controls by higher proportions of ARA, 22:6n-3 (DHA) and total PUFAs, elevated DBIs and lower percentages of 17:0 and 18:1n-9.

Knee OA was featured by reduced levels of 14:0, 14:1n-5, 16:1n-5, 17:0*ai*, LA, 22:0, 24:0, n-6 PUFAs and total PUFAs compared with the control or RA knees. The proportions of 14:0, 14:1n-5, 15:0, 16:1n-5, 17:0*ai* and 22:0 were elevated and those of LA reduced together with decreased TACLs in the RA knees compared with the corresponding controls. Within a diagnosis, shoulder OA had higher percentages of LA, DHA, 24:0, n-6 PUFAs and total PUFAs compared with knee OA while the opposite was true for 17:0*ai*. Regarding RA, knees displayed higher levels of 14:0, 14:1n-5, 15:0, 16:1n-5, 17:0*ai*, 17:0, 18:1n-9, 18:1n-7, 22:1n-9 and total MUFAs than shoulders. The proportions of LA, ARA, DHA, n-6 PUFAs and total PUFAs were lower together with reduced DBIs and TACLs in knees.

**Figure 4:**
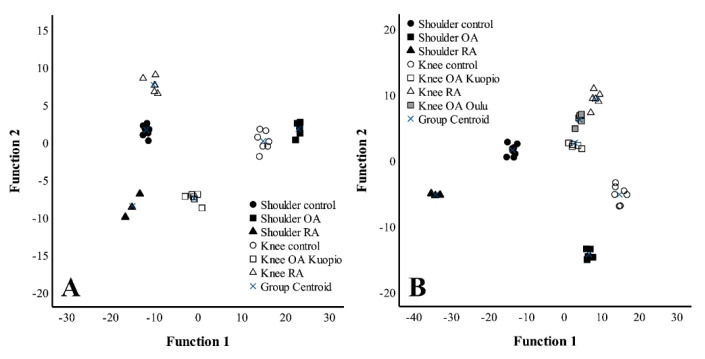
Discriminant analyses depicting the classification of fatty acid signatures in synovial fluids. (**A**) OA knee samples were only included from the Kuopio subgroup, (**B**) OA knee subgroups were from both Kuopio and Oulu. OA = osteoarthritis, RA = rheumatoid arthritis. Note that the scaling is different in the x- and y-axes and between the panels.

The discriminant analysis was performed first without the Oulu OA knee population. All six study groups were clearly separated from each other based on their FA signatures (Figure 4A). The variables with the largest separation power included 22:5n-3, DHA and 15:0. With the first two functions, 93.7% of the variance was explained and the analysis classified 100% of the samples correctly into their respective study groups. When the Oulu OA knees were included in the analysis, they clustered next to the Kuopio OA knees and the RA knees (Figure 4B). In this case, LA, 18:0 and 22:1n-9 were the variables with the largest separation power and 92.3% of the variance was explained by functions 1–2. The model continued to predict 100% of the samples correctly.

There were no statistically significant correlations between the age, body mass or BMI and the proportions of the most abundant FAs and the FA sums. In fact, 24:0, a relatively minor SFA, was the only FA with significant differences based on the diagnosis and joint, which also showed a weak but significant relationship with age (r_s_ = −0.378, *p* = 0.014). The FA data were also analyzed with partial correlations adjusted for age and BMI and the only significant correlation was found between age and 24:0 when adjusted for BMI (r = −0.394, *p* = 0.012).

## 4. Discussion

The current literature provides evidence of simultaneous and complex pro-inflammatory and pro-resolving processes being present in OA and RA joints [3,12] and it is plausible that shifts in the balance of these cascades determine the progression of joint destruction. PUFAs such as ARA give rise to numerous lipid mediators with a diverse potential to affect joint health [3,12]. This is the pivotal issue that makes the interpretation of FA profiles complicated in inflammatory conditions as manifestations of both pro-inflammatory and resolving kinds can be observed at the same time. Little is known about the similarity of SF FA composition between different joints in health and disease even though individual FAs have been demonstrated to have diverse effects on tissues comprising synovial joints and they may potentially contribute to the pathogeneses of joint diseases [13]. In the present study, the FA signatures of control (trauma) shoulders and knees were found to be very uniform. Only the percentages of 17:0, EPA and 22:1n-9, all FAs of minor proportion, were significantly higher in knees despite the different weight-bearing properties, stability and mobility of these joints [27,28].

Another finding that calls for explanation was the higher proportion of individual and total PUFAs in shoulders compared with knees in the diseased states. A recent study by Chubinskaya et al. [32] suggested that the intrinsic reparative capacity of the shoulder articular cartilage could be limited when subjected to inflammatory conditions. This may be associated with the poor healing ability of the glenohumeral joint and its vulnerability to surgical interventions. In this scenario, the elevated SF PUFA proportions could be considered beneficial leading to reduced friction in the shoulder joint and they could participate in the process of the resolution of inflammation. On the other hand, the knee as a weight-bearing joint could be generally expected to experience more wear and tear unless there is overt trauma to the shoulder joint. For this reason, the knee could be assumed to benefit more from increased lubrication provided by SF PUFAs but, in reality, the results of the knee joint showed lower PUFA percentages. Hypothetical contributors to location-dependent PUFA profiles could be different joint surface areas, SF volumes, permeabilities of joint capsule and secretion of lipids by infrapatellar fat pad (IFP) but the elucidation of their potential roles would require, for example, detailed analyses of FA metabolic pathways in future studies. Previously, the IFP of end-stage OA was noted to secrete ARA and DHA into fat-conditioned medium [35] but, to the best of our knowledge, the FAs preferentially released from IFP into SF in health and disease remain unknown. If IFP complies with the selective patterns of FA mobilization documented for other adipose tissues [36], the levels of, for instance, 20C PUFAs could be expected to increase in the SF of the arthritic knee, which was not observed in the present study.

RA shoulders were characterized by elevated percentages of ARA and DHA and with reduced 18:1n-9 proportions compared with control shoulders. These novel results are probably also reflections of the simultaneous inflammatory and pro-resolving processes present in the diseased joint [3,12]. Previously, ARA levels in knee SFs were not altered in RA patients compared to those with OA [8] but the results of the present experiment gain support from the literature showing elevated levels of ARA-derived lipid mediators in RA SF [12]. In respect to knee/hip OA, ARA was previously elevated or reduced in SF [11,37], accumulated in articular cartilage and cancellous bone [7,38] and its levels in plasma phospholipids (PLs) correlated with synovitis [39]. In cell cultures, ARA and its precursor, LA, can increase the markers of inflammation and cartilage degradation [1,23,40,41]. However, ARA can also induce beneficial effects on synoviocytes, cartilage and bone via PGE_2_, 15-deoxy-∆^12,14^-prostaglandin J_2_ and lipoxin A_4_ (LXA_4_) [19,20,42,43]. For instance, PGE_2_ may have a dual role in pannus formation in RA [21,22].

DHA functions as an anti-inflammatory PUFA and a precursor for D-series resolvins, protectins and maresins [44]. Its elevated SF proportions in RA shoulders could participate in the joint’s attempt to resolve inflammation and, consequently, to prevent progressive cartilage/bone damage. In fact, in vitro studies and in vivo animal experiments have documented reduced arthritis symptoms, less cartilage damage, increased articular cartilage thickness and anabolic effects on bone by DHA alone or together with EPA [19,26,45]. In humans, a high intake of these long-chain n-3 PUFAs may protect from the development of RA and alleviate related pain and other symptoms [26]. DHA-derived lipid mediators were previously elevated in SF from RA knees [12] and their hypothetically increased production could counteract the inflammatory processes in RA shoulders. In respect to dietary MUFAs, especially 18:1n-9 has been suggested to play a potential role in the prevention of RA and in the reduction of disease activity [46,47]. In the present experiment, 18:1n-9 proportions decreased in RA shoulders while a previous study documented increased levels of 20–24C MUFAs in RA SF [8]. However, a higher intake of dietary MUFAs tended to be associated with reduced radiographic progression of knee OA [24]. In cell cultures, the effects of 18:1n-9 have been conflicting: increased cytokine release and chondrocyte apoptosis or decreased 16:0-induced lipotoxicity and cartilage destruction [1,41,48].

Regarding other MUFAs, shoulder OA was characterized by elevated proportions of 18:1n-7. Previously, the higher plasma PL levels of this MUFA were associated, for instance, with a lower risk of heart failure with antecedent coronary heart disease [49]. Regarding joint diseases, elevated 18:1n-7 levels were documented in the plasma PLs of RA patients compared with controls but not in RA SF vs. OA SF [8]. This particular MUFA has not been determined in related studies on humans and its role in arthritis remains unknown. In contrast to some previous studies, we did not observe significant changes in the proportions of major SFAs in the diseased SFs. For instance, 16:0 and 18:0, which have been associated with arthritic processes [14,15,16], remained unresponsive in the present study. Regarding the knee joint, 14:0 percentages were observed to be elevated in RA. This SFA can have both beneficial and detrimental effects on joints but, generally, its influence has been more protective than harmful compared with those of 16:0 and 18:0 [16]. To sum up, the observed FA alterations in RA shoulders include potentially beneficial (increased DHA) but also detrimental changes (elevated ARA, reduced 18:1n-9) and probably reflect the simultaneous inflammatory and pro-resolving processes present in the diseased joint.

The FA profiles of OA and RA knees were relatively similar and featured lower proportions of LA, DHA and total n-6 PUFAs compared with trauma controls or shoulders with the same diagnosis. The reduced n-6 PUFA levels in the diseased knees may seem surprising due to the inflammatory role that especially ARA-derivatives play but there is accumulating evidence for an inverse association between arthritis and LA or ARA levels in circulation or in SF [8,11,50,51,52]. It remains unresolved if the reduction in n-6 PUFAs results in a decreased inflammatory load or whether it is a consequence of their intensified conversion to lipid mediators. Due to the higher loading of the knee joint, it would be plausible that its tissues allocated more PUFAs to oxylipin synthesis in inflammatory conditions than those of the less-loaded shoulder joint. Previously, He et al. [53] reported decreased levels of LA-derived lipid mediators in the plasma of mice with collagen-induced arthritis while several serum n-6 PUFAs correlated positively with OA severity and/or synovitis in a murine OA model [25]. In addition, postprandial plasma n-6 PUFA levels were associated with structural knee OA and joint effusion in men [54]. The case of the pro-inflammatory ARA-derived PGE_2_ is complex as it can also contribute to the resolution of inflammation by modulating the production of LXA_4_ [55].

Compared with OA shoulders, OA knees also showed decreased proportions of DHA—a long-chain n-3 PUFA capable of inducing beneficial effects on chondrocytes and synoviocytes [17,18]. DHA levels in plasma PLs were negatively associated with patellofemoral cartilage loss in human subjects [39] and SF DHA concentrations correlated inversely with OA severity in mice [25]. Together with the present data, these results suggest that there would be impaired resolution of inflammation in OA knees via an insufficient production of SPMs from DHA. Moreover, total PUFA percentages were lower in the SF of both OA and RA knees compared with knee controls and/or shoulders with the same diagnosis. These findings endorse the previous study by Lu et al. [24] who documented that higher amounts of PUFAs in self-reported diets were associated with reduced radiographic progression of knee OA. To summarize, decreased n-6 PUFA and DHA proportions may result in simultaneously beneficial and detrimental processes in the diseased knee joints and be reflected in the profiles of their downstream lipid mediators.

SF lipids can be filtrated from plasma, be produced and released locally or derive from the destruction of joint tissues [35,56,57]. Only 0.2% of SF consists of lipids and 33% of them are PLs [58]. Approximately 67% of these PLs are phosphatidylcholine (PC) with increased levels in OA and RA, both of which can also be characterized by elevated proportions of longer PC species but reduced percentages of saturated PC species in SF [59]. The RA shoulder data of the present study, with the increased proportions of particular long-chain PUFAs together with the highest TACL and DBI values, were compatible with this earlier literature and may be related to reduced friction [59] but the FA alterations in OA and RA knees were the opposite. In addition, RA shoulders did not accumulate long-chain SFAs and 24:1n-9 as could have been expected due to increased sphingomyelin levels in OA and RA SF [59,60].

The present study has limitations that should be considered. Due to the small number of samples available, the patient groups could not be matched for age, gender or BMI. Thus, the documented differences in the FA profiles between the controls and OA/RA patients can partly derive from the fact that the latter groups mostly consisted of women that were older and tended to be heavier (OA) or thinner (RA) than the trauma controls. Normal aging can influence the FA composition of tissues and it has been shown, for instance, that ARA and DHA levels decrease in cortical grey matter in the elderly [61]. Gender distribution could also present a confounding factor as sex hormones may influence the activities of enzymes involved in long-chain PUFA synthesis [62]. In this respect, the levels of ARA and DHA were documented to be higher in the plasma total lipids and PLs of women compared with men. When the present FA data were analyzed with partial correlations adjusted for age and BMI, it could be observed that there were very few weak but significant correlations between these potentially confounding factors and the FA mol-% and that these FAs were not among the most abundant. Moreover, the number of SF samples from RA shoulders was limited and these results need to be confirmed in future studies.

## 5. Conclusions

The results of the present study indicate location- and disease-dependent differences in the SF FA profiles of shoulders and knees. Against expectations, the RA FA signatures were not clearly more inflammatory than those of the OA SF in either joint. The FA profiles of control shoulders and knees were mostly similar, despite the very different weight-bearing nature and range of motion of these joints. However, the two joints reacted differently to inflammatory conditions as OA and RA shoulders were characterized by elevated PUFA proportions compared with OA and RA knees. It remains to be determined if and how the characteristics of the two joints would be causative factors in this phenomenon. The observed FA alterations in OA and RA may contribute to the pathogeneses of inflammatory joint diseases in addition to participating in the resolution pathways and they support earlier literature indicating that simultaneous pro-inflammatory and pro-resolving processes would be present in OA and RA joints.

## Figures and Tables

**Table 1:** General characteristics of the patients (mean ± SE).

Group	Control	OA	RA	*p*	*p*	*p*
				Diagnosis	Location	Interaction
Sex (M/F)	9/7	4/13	1/8	0.053		
Age	44 ± 3	62 ± 2 *	71 ± 3 *^,†^	0.020	0.021	0.002
Body mass	80.2 ± 3.86	87.6 ± 4.54	65.6 ± 4.57	0.170	0.926	0.592
BMI	26.8 ± 1.13	31.7 ± 1.55	25.2 ± 1.09	0.700	0.509	0.925

OA = osteoarthritis, RA = rheumatoid arthritis, M = male, F = female, BMI = body mass index. Sex ratios were tested with the Fisher’s exact test. * = significant difference from control, ^†^ = significant difference from OA (generalized linear model, *p* < 0.05).

## Data Availability

All relevant data generated or analyzed during this study are included in this published article and its supplement.

## Supplementary Materials

The following is available online at https://www.mdpi.com/2079-7737/10/5/401/s1, Table S1: Fatty acid composition of the synovial fluid in the shoulder and knee joints based on diagnosis (mean ± SE).

## Abbreviations

ARA arachidonic acid, BMI body mass index, DBI double bond index, DHA docosahexaenoic acid, EPA eicosapentaenoic acid, FA fatty acid, FAME fatty acid methyl ester, IFP infrapatellar fat pad, LA linoleic acid, LXA_4_ lipoxin A_4_, MUFA monounsaturated fatty acid, OA osteoarthritis, PC phosphatidylcholine, PGE_2_ prostaglandin E_2_, PL phospholipid, PUFA polyunsaturated fatty acid, RA rheumatoid arthritis, r_s_ Spearman correlation coefficient, SF synovial fluid, SFA saturated fatty acid, SPM specialized pro-resolving mediator, TACL total average chain length.
