# Supplementary material for: Synovial Fluid Fatty Acid Profiles Are Differently Altered by Inflammatory Joint Pathologies in the Shoulder and Knee Joints
Source: Biology (Basel). 2021 May 4;10(5):401. doi: 10.3390/biology10050401 (PMC8147852; doi:10.3390/biology10050401)
Supplement: Supplementary file 1 [file biology-10-00401-s001.zip › biology-1188004-supplementary.pdf]

**Supplementary Table 1.** Fatty acid composition of the synovial fluid in the shoulder and knee joints based on diagnosis (mean ± SE).

| Fatty acid | Shoulder Control | Shoulder OA | Shoulder RA | Knee Control | Knee OA | Knee RA | $P^a$ | $P^b$ | $P^c$ |
|---|---|---|---|---|---|---|---|---|---|
| 14:0 | 3.371 ± 0.238 | 3.183 ± 0.319 | 2.851 ± 0.292 | 3.412 ± 0.252 | 3.524 ± 0.181 | 4.110 ± 0.116 | 0.884 | 0.005 | 0.050 |
| 14:1n-5 | 0.257 ± 0.036 | 0.260 ± 0.029 | 0.227 ± 0.023 | 0.251 ± 0.040 | 0.309 ± 0.026 | 0.415 ± 0.052 | 0.231 | 0.013 | 0.050 |
| 15:0 | 0.237 ± 0.017 | 0.206 ± 0.021 | 0.181 ± 0.023 | 0.232 ± 0.011 | 0.256 ± 0.019 | 0.276 ± 0.031 | 0.959 | 0.009 | 0.072 |
| DMA 16:0 | 0.080 ± 0.016 | 0.091 ± 0.024 | 0.076 ± 0.020 | 0.091 ± 0.016 | 0.090 ± 0.016 | 0.092 ± 0.008 | 0.930 | 0.554 | 0.898 |
| 16:0 | 28.957 ± 0.620 | 28.181 ± 0.716 | 27.932 ± 0.256 | 28.243 ± 0.653 | 29.190 ± 0.648 | 29.159 ± 0.596 | 0.981 | 0.382 | 0.275 |
| 16:1n-9 | 0.381 ± 0.035 | 0.410 ± 0.037 | 0.405 ± 0.015 | 0.399 ± 0.027 | 0.392 ± 0.026 | 0.420 ± 0.020 | 0.782 | 0.835 | 0.803 |
| 16:1n-7 | 2.248 ± 0.152 | 2.771 ± 0.736 | 2.822 ± 0.297 | 2.157 ± 0.183 | 3.348 ± 0.556 | 2.847 ± 0.554 | 0.152 | 0.686 | 0.747 |
| 16:1n-5 | 0.198 ± 0.031 | 0.211 ± 0.050 | 0.179 ± 0.043 | 0.207 ± 0.027 | 0.228 ± 0.026 | 0.345 ± 0.039 | 0.265 | 0.025 | 0.076 |
| 17:0*i* | 0.122 ± 0.034 | 0.101 ± 0.026 | 0.071 ± 0.019 | 0.101 ± 0.007 | 0.091 ± 0.015 | 0.124 ± 0.027 | 0.748 | 0.727 | 0.344 |
| 17:0*ai* | 0.262 ± 0.042 | 0.197 ± 0.023 | 0.179 ± 0.037 | 0.238 ± 0.027 | 0.275 ± 0.017 | 0.359 ± 0.037 | 0.621 | 0.003 | 0.008 |
| 17:0 | 0.470 ± 0.036 | 0.452 ± 0.064 | 0.319 ± 0.011 | 0.576 ± 0.046 | 0.525 ± 0.047 | 0.559 ± 0.041 | 0.270 | 0.001 | 0.269 |
| 17:1n-8 | 0.168 ± 0.015 | 0.190 ± 0.024 | 0.153 ± 0.042 | 0.154 ± 0.021 | 0.168 ± 0.012 | 0.179 ± 0.018 | 0.539 | 0.833 | 0.475 |
| DMA 18:0 | 0.285 ± 0.044 | 0.289 ± 0.011 | 0.336 ± 0.019 | 0.332 ± 0.031 | 0.289 ± 0.034 | 0.390 ± 0.028 | 0.192 | 0.286 | 0.733 |
| DMA 18:1n-9 | 0.252 ± 0.048 | 0.264 ± 0.064 | 0.179 ± 0.026 | 0.266 ± 0.053 | 0.239 ± 0.041 | 0.375 ± 0.052 | 0.901 | 0.151 | 0.124 |
| DMA 18:1n-7 | 0.050 ± 0.011 | 0.058 ± 0.022 | 0.055 ± 0.013 | 0.030 ± 0.003 | 0.056 ± 0.008 | 0.056 ± 0.008 | 0.157 | 0.427 | 0.565 |
| 18:0 | 13.317 ± 0.908 | 12.836 ± 1.096 | 11.224 ± 0.930 | 13.610 ± 0.714 | 14.513 ± 1.173 | 14.768 ± 1.071 | 0.859 | 0.052 | 0.401 |
| 18:1n-9 | 23.866 ± 0.720 | 23.924 ± 1.228 | 21.261 ± 0.545 | 23.762 ± 1.146 | 26.182 ± 1.119 | 25.739 ± 0.998 | 0.337 | 0.018 | 0.146 |
| 18:1n-7 | 2.466 ± 0.114 | 2.971 ± 0.287 | 2.685 ± 0.162 | 2.763 ± 0.133 | 2.977 ± 0.103 | 2.995 ± 0.075 | 0.017 | 0.084 | 0.449 |
| 18:1n-5 | 0.682 ± 0.148 | 0.706 ± 0.153 | 0.489 ± 0.080 | 0.630 ± 0.061 | 0.586 ± 0.081 | 0.922 ± 0.117 | 0.893 | 0.377 | 0.077 |
| 18:2n-7 | 0.210 ± 0.042 | 0.250 ± 0.036 | 0.185 ± 0.035 | 0.278 ± 0.030 | 0.239 ± 0.021 | 0.231 ± 0.042 | 0.566 | 0.233 | 0.445 |
| 18:2n-6 | 13.062 ± 1.826 | 14.842 ± 2.209 | 17.581 ± 1.254 | 13.829 ± 2.120 | 9.511 ± 1.096 | 8.300 ± 0.878 | 0.703 | 0.001 | 0.012 |
| 18:2*c*9*t*11 | 0.079 ± 0.021 | 0.083 ± 0.029 | 0.066 ± 0.025 | 0.091 ± 0.026 | 0.066 ± 0.010 | 0.077 ± 0.011 | 0.775 | 0.911 | 0.677 |
| 18:3n-6 | 0.081 ± 0.032 | 0.067 ± 0.024 | 0.044 ± 0.020 | 0.065 ± 0.020 | 0.065 ± 0.013 | 0.148 ± 0.075 | 0.719 | 0.315 | 0.227 |
| 18:3n-3 | 0.605 ± 0.051 | 0.540 ± 0.157 | 0.662 ± 0.075 | 0.598 ± 0.053 | 0.556 ± 0.037 | 0.649 ± 0.046 | 0.308 | 0.976 | 0.972 |
| 19:1n-8 | 0.065 ± 0.018 | 0.075 ± 0.032 | 0.046 ± 0.015 | 0.072 ± 0.018 | 0.067 ± 0.008 | 0.076 ± 0.014 | 0.855 | 0.514 | 0.586 |
| 20:0 | 0.381 ± 0.100 | 0.245 ± 0.016 | 0.273 ± 0.068 | 0.280 ± 0.032 | 0.250 ± 0.019 | 0.236 ± 0.013 | 0.222 | 0.360 | 0.589 |
| 20:1n-11 | 0.708 ± 0.413 | 0.095 ± 0.011 | 0.232 ± 0.178 | 0.149 ± 0.037 | 0.196 ± 0.047 | 0.094 ± 0.008 | 0.315 | 0.290 | 0.256 |
| 20:1n-9 | 0.275 ± 0.139 | 0.127 ± 0.015 | 0.130 ± 0.038 | 0.321 ± 0.138 | 0.222 ± 0.029 | 0.198 ± 0.039 | 0.265 | 0.387 | 0.960 |
| 20:1n-7 | 0.228 ± 0.159 | 0.027 ± 0.007 | 0.124 ± 0.093 | 0.125 ± 0.041 | 0.092 ± 0.022 | 0.103 ± 0.010 | 0.345 | 0.795 | 0.576 |
| 20:2n-9 | 0.357 ± 0.247 | 0.041 ± 0.011 | 0.157 ± 0.091 | 0.159 ± 0.092 | 0.129 ± 0.056 | 0.112 ± 0.025 | 0.393 | 0.669 | 0.543 |
| 20:2n-6 | 0.173 ± 0.093 | 0.114 ± 0.040 | 0.079 ± 0.026 | 0.212 ± 0.034 | 0.146 ± 0.020 | 0.164 ± 0.023 | 0.340 | 0.255 | 0.901 |
| 20:3n-6 | 0.791 ± 0.129 | 0.875 ± 0.099 | 0.746 ± 0.356 | 0.743 ± 0.116 | 0.616 ± 0.057 | 0.590 ± 0.062 | 0.708 | 0.102 | 0.587 |
| 20:4n-6 | 2.892 ± 0.418 | 2.741 ± 0.449 | 4.889 ± 0.389 | 2.989 ± 0.530 | 2.325 ± 0.313 | 2.092 ± 0.265 | 0.107 | 0.003 | 0.004 |
| 20:4n-3 | 0.677 ± 0.137 | 0.637 ± 0.172 | 0.746 ± 0.110 | 0.626 ± 0.049 | 0.636 ± 0.065 | 0.821 ± 0.093 | 0.412 | 0.931 | 0.859 |
| 20:5n-3 | 0.116 ± 0.044 | 0.237 ± 0.050 | 0.163 ± 0.116 | 0.377 ± 0.085 | 0.256 ± 0.061 | 0.285 ± 0.058 | 0.945 | 0.017 | 0.136 |
| 22:0 | 0.164 ± 0.033 | 0.199 ± 0.034 | 0.170 ± 0.044 | 0.203 ± 0.018 | 0.216 ± 0.027 | 0.335 ± 0.086 | 0.307 | 0.035 | 0.241 |
| 22:1n-11 | 0.044 ± 0.008 | 0.038 ± 0.004 | 0.047 ± 0.005 | 0.042 ± 0.006 | 0.042 ± 0.004 | 0.048 ± 0.008 | 0.583 | 0.873 | 0.917 |
| 22:1n-9 | 0.055 ± 0.010 | 0.079 ± 0.009 | 0.072 ± 0.014 | 0.123 ± 0.009 | 0.096 ± 0.013 | 0.135 ± 0.020 | 0.525 | 0.000019 | 0.097 |
| 22:1n-7 | 0.026 ± 0.006 | 0.031 ± 0.007 | 0.026 ± 0.006 | 0.027 ± 0.004 | 0.031 ± 0.006 | 0.027 ± 0.005 | 0.679 | 0.953 | 0.990 |
| 22:4n-6 | 0.104 ± 0.058 | 0.048 ± 0.009 | 0.035 ± 0.012 | 0.041 ± 0.009 | 0.049 ± 0.005 | 0.030 ± 0.004 | 0.457 | 0.391 | 0.488 |
| 22:5n-3 | 0.106 ± 0.032 | 0.053 ± 0.012 | 0.043 ± 0.008 | 0.075 ± 0.012 | 0.134 ± 0.038 | 0.149 ± 0.044 | 0.990 | 0.085 | 0.115 |
| 22:6n-3 | 0.613 ± 0.063 | 0.759 ± 0.071 | 0.900 ± 0.173 | 0.599 ± 0.087 | 0.411 ± 0.061 | 0.470 ± 0.054 | 0.463 | 0.00003 | 0.011 |
| 24:0 | 0.393 ± 0.018 | 0.391 ± 0.040 | 0.361 ± 0.011 | 0.380 ± 0.019 | 0.288 ± 0.015 | 0.335 ± 0.027 | 0.045 | 0.010 | 0.058 |
| 24:1n-9 | 0.084 ± 0.021 | 0.080 ± 0.017 | 0.077 ± 0.005 | 0.106 ± 0.014 | 0.089 ± 0.013 | 0.134 ± 0.023 | 0.569 | 0.052 | 0.463 |
| SFA | 47.673 ± 1.478 | 45.992 ± 2.199 | 43.559 ± 1.493 | 47.274 ± 1.637 | 49.127 ± 1.958 | 50.261 ± 1.763 | 0.951 | 0.055 | 0.224 |
| MUFA | 31.750 ± 0.871 | 31.996 ± 2.010 | 28.976 ± 0.202 | 31.287 ± 1.431 | 35.027 ± 1.669 | 34.677 ± 1.593 | 0.363 | 0.041 | 0.177 |
| PUFA | 19.911 ± 1.991 | 21.311 ± 2.636 | 26.818 ± 1.602 | 20.721 ± 2.890 | 15.173 ± 1.406 | 14.149 ± 1.136 | 0.460 | 0.001 | 0.008 |
| n-6 PUFA | 17.103 ± 2.212 | 18.686 ± 2.704 | 23.375 ± 1.303 | 17.879 ± 2.747 | 12.712 ± 1.393 | 11.800 ± 0.920 | 0.571 | 0.001 | 0.016 |
| n-3 PUFA | 2.116 ± 0.131 | 2.226 ± 0.289 | 2.514 ± 0.279 | 2.274 ± 0.165 | 1.993 ± 0.087 | 3.276 ± 0.936 | 0.098 | 0.438 | 0.433 |
| n-9 PUFA | 0.402 ± 0.266 | 0.066 ± 0.020 | 0.679 ± 0.434 | 0.200 ± 0.091 | 0.162 ± 0.061 | 0.547 ± 0.398 | 0.105 | 0.659 | 0.734 |
| DMA | 0.666 ± 0.103 | 0.702 ± 0.107 | 0.646 ± 0.053 | 0.718 ± 0.086 | 0.673 ± 0.085 | 0.758 ± 0.143 | 0.992 | 0.613 | 0.822 |
| UFA/SFA | 1.100 ± 0.065 | 1.179 ± 0.106 | 1.286 ± 0.078 | 1.115 ± 0.070 | 1.061 ± 0.089 | 1.027 ± 0.066 | 0.868 | 0.098 | 0.328 |
| n-3/n-6 PUFA | 0.157 ± 0.035 | 0.131 ± 0.026 | 0.109 ± 0.014 | 0.148 ± 0.024 | 0.181 ± 0.026 | 0.301 ± 0.100 | 0.508 | 0.308 | 0.109 |
| prod/prec n-6 | 0.289 ± 0.025 | 0.245 ± 0.012 | 0.324 ± 0.023 | 0.267 ± 0.010 | 0.310 ± 0.023 | 0.321 ± 0.016 | 0.131 | 0.469 | 0.086 |
| prod/prec n-3 | 1.302 ± 0.171 | 1.454 ± 0.229 | 1.657 ± 0.479 | 1.698 ± 0.329 | 1.268 ± 0.180 | 1.192 ± 0.173 | 0.822 | 0.675 | 0.181 |
| DBI | 0.836 ± 0.041 | 0.869 ± 0.056 | 1.001 ± 0.045 | 0.852 ± 0.062 | 0.755 ± 0.037 | 0.735 ± 0.030 | 0.521 | 0.002 | 0.017 |
| TACL | 17.289 ± 0.042 | 17.270 ± 0.044 | 17.361 ± 0.037 | 17.281 ± 0.050 | 17.204 ± 0.029 | 17.150 ± 0.022 | 0.402 | 0.004 | 0.055 |

[a]between diagnoses, [b]between locations, [c]diagnosis × location interaction (generalized linear model, $p<0.05$)

OA = osteoarthritis, RA = rheumatoid arthritis, DMA = dimethyl acetal, *i.e.*, plasmalogen alkenyl chain derivative, *i* = iso, *ai* = anteiso, *c* = cis, *t* = trans, SFA = saturated fatty acid, MUFA = monounsaturated fatty acid, PUFA = polyunsaturated fatty acid, UFA = unsaturated fatty acid (MUFA + PUFA), prod = product, prec = precursor, DBI = double bond index, TACL = total average chain length
